# Estimation of leaf water content from hyperspectral data of different plant species by using three new spectral absorption indices

**DOI:** 10.1371/journal.pone.0249351

**Published:** 2021-03-30

**Authors:** Hong Li, Wunian Yang, Junjie Lei, Jinxing She, Xiangshan Zhou

**Affiliations:** 1 College of Earth Science, Chengdu University of Technology, Chengdu, China; 2 Geology and Surveying Engineering School, Chongqing Vocational Institute of Engineering, Chongqing, China; Universidade Federal de Uberlandia, BRAZIL

## Abstract

The leaf equivalent water thickness (EWT, g cm^−2^) and fuel moisture content (FMC, %) are key variables in ecological and environmental monitoring. Although a variety of hyperspectral vegetation indices have been developed to estimate the leaf EWT and FMC, most of these indices are defined considered two or three specific bands for a specific plant species, which limits their applicability. In this study, we proposed three new spectral absorption indices (SAI_970_, SAI_1200_, and SAI_1660_) for various plant types by considering the symmetry of the spectral absorption at 970 nm, 1200 nm and 1660 nm and spectral heterogeneity of different leaves. The indices were calculated considering the absorption peak and shoulder bands of each leaf instead of the same specific bands for all leaves. A pooled dataset of three tree species (camphor (VX), capricorn (VJ), and red-leaf plum (VL)) was used to test the performance of the SAIs in terms of the leaf EWT and FMC estimation. The results indicated that, first, SAI_1200_ was more suitable for estimating the EWT than FMC, whereas SAI_970_ and SAI_1660_ were more suitable for estimating the FMC. Second, SAI_1200_ achieved the most accurate estimation of the EWT with a cross-validation coefficient of determination (*R*_*cv*_^*2*^) of 0.845 and relative cross-validation root mean square error (*rRMSE*_*cv*_) of 8.90%. Third, SAI_1660_ outperformed the other indices in estimating the FMC at the leaf level, with an *R*_*cv*_^*2*^ of 0.637 and *rRMSE*_*cv*_ of 8.56%. Fourth, SAI_970_ achieved a moderate accuracy in estimating the EWT (*R*_*cv*_^*2*^ of 0.25 and *rRMSE*_*cv*_ of 19.68%) and FMC (*R*_*cv*_^*2*^ of 0.275 and *rRMSE*_*cv*_ of 12.10%) at the leaf level. These results can enrich the application of the SAIs and demonstrate the potential of using SAI_1200_ to determine the leaf EWT and SAI_1660_ to obtain the leaf FMC among various plant types.

## Introduction

The vegetation water content (VWC) is a valuable indicator of vegetation drought stress [1], forest fire risk [2, 3], and regional water resource assessment [4]. The most accurate method to evaluate the water status of vegetation involves traditional physiological measurements; however, this method is time consuming, laborious, and cannot meet the requirements of large-scale, real-time monitoring [5]. The development of remote sensing technology has enabled the prompt monitoring of the VWC in real time in a nondestructive manner over a large area [6]. The accurate evaluation of the VWC based on spectral reflectance measurements has always been a key research topic in remote sensing [7–15]. The main VWC parameters are the leaf level equivalent water thickness (EWT, g cm^−2^), which is based on area, and the fuel moisture content (FMC), which is based on mass [16]. Most studies have aimed to develop techniques for or evaluate the use of the spectral reflectance to estimate the EWT [17–21]. Moreover, certain studies indicated that the spectral reflectance is highly correlated with the FMC [22–25].

At present, the main methods used to estimate the leaf water content based on statistical analysis include spectral indices [26–31], derivative spectra [24, 32] and post continuum removal indicators [33–35]. In this work, we focused on only the spectral indices. The vegetation spectral index is widely used to analyze the vegetation biophysical properties because of its simplicity and high generalizability. In the spectral domain (400–2500 nm), water absorption features, which appear at approximately 1200 nm, 970 nm, 1950 nm and 1450 nm [36, 37], are generally used to estimate the VWC [21, 38–40]. Moreover, certain studies reported a strong correlation between the reflectance spectra between 1650 and 1850 nm and water content of leaves [25, 36]. Based on these specific absorption values of water occurring across localized spectral regions of short wave infrared (SWIR) and near infrared (NIR) bands, many different vegetation water diagnostic indicators have been proposed, such as the water index (WI) [41], simple ratio water index (SRWI) [42], moisture stress index (MSI) [43], three-band ratio indices (RATIO_975_ and RATIO_1200_) [24], normalized difference water index (NDWI) [10], normalized difference infrared index (NDII) [44], global vegetation moisture index (GVMI) [45], relative depth index (RDI) [32], and depth water index (DWI) [46]. Although these indices have been successfully used to estimate the EWT and FMC at the leaf or canopy levels [35, 42–44, 47, 48], the indices were developed only considering two or three specific bands (e.g., the WI, SRWI, MSI, NDWI, NDII, GVMI) or to examine a specific plant species. For example, Pu et al. [29] used two three-band ratio indices (RATIO_975_ and RATIO_1200_) to assess the water status (leaf FMC) of oak leaves by considering the water absorption characteristics in the range of 920–1110 nm and 1090–1285 nm, respectively. Delegido Pasqualotto et al. [46] proposed the DWI to accurately predict the EWT at the canopy level within regions covered by different crop types. However, the DWI was calculated considering only four specific bands for all leaves.

The indices proposed in the abovementioned studies were calculated considering the same specific bands for all leaves, and the differences in the spectral absorption characteristics among different leaves, especially those of different vegetation species were mostly ignored. However, the contents of water and other components vary among different leaves, and this phenomenon may lead to the location deviation of the peaks or troughs of the spectral absorption features. Spectral absorption characteristics represent a valuable tool to study the composition and content of substances by remote sensing technology. Previous studies have shown that the spectral absorption index (SAI), which was first proposed by Wang et al. [49] and applied to remote sensing geology, can reflect the variation characteristics of the spectral absorption features [50]. To realize mineral mapping, Wang et al. [49] defined certain SAIs and used SAI_2175_ and SAI_2295_ to extract alteration tuff and altered basalt information, respectively, from far-ultraviolet imaging spectrograph images in the Hatu Mining Area. Recently, Li et al. [51] reported that SAI_680_ was the most sensitive to changes in the fraction of absorption photosynthetically active radiation (FAPAR) compared to those in the absorption peak depth (ad), absorption peak symmetry (AA) and NDVI. These previous studies indicated that more accurate retrieval results could be obtained using SAIs owing to the consideration of the continuous reflectance curve characteristics of different leaves rather instead of several specific bands for all leaves. However, to date, the application of SAI in estimating the VWC has not been reported. Therefore, this study aims to develop a new SAI that not only considers the spectral reflectance heterogeneity of different leaves but can also be used for various plant species. Moreover, the accuracies of the new SAIs and abovementioned ten typical spectral indices in estimating the leaf EWT and FMC are compared.

## Materials and methods

### Data collection

The study area was located in the Chengdu University of Technology, Sichuan Province, China. Three species of trees (camphor (VX), capricorn (VJ), red-leaf plum (VL)) were selected for leaf sampling in the sample area under clear and cloudless weather on May 15, 2019. Overall, 292 leaf samples in the tree crowns were collected. The capricorn (VJ) and red-leaf plum (VL) trees had only new leaves, while the camphor tree (VX) had both new and mature leaves. The collected samples were immediately sealed in plastic bags and transported to the laboratory in a cooler at 5°C.

### Water content and leaf reflectance measurements

Under controlled laboratory conditions, leaf weight and spectral reflectance measurements were collected to reduce any possible error caused by changing atmospheric conditions. To avoid the influence of high temperatures on the water content of leaves during the contact spectrum measurement, the fresh weight (FW, g) of each leaf was first measured. Next, the spectral data of each leaf were obtained using a FieldSpec^®^ 3 field spectroradiometer (ASD, Inc.; Boulder, Colorado) with a wide spectral range (350 to 2500 nm). During the spectral measurement process, blackout curtains were used to shade the window, and the leaves were placed on black flannelette to ensure that the only light source pertained to the measurement instrument. The spectral values of the upper, middle and lower parts of each leaf were measured, and the mean of the three measurements was considered as the spectral value of the leaf. Next, the leaves were scanned, and digital processing was conducted in MapGIS to obtain the area of each leaf polygon (A, cm^2^). Finally, all the leaves were dried at 80°C to obtain the dry weight (DW, g).

### Calculation of the vegetation water indices

The EWT [33] and FMC were calculated using Eqs (1) and (2), respectively.

$$EWT=\frac{FW-DW}{A},$$ (1)

$$FMC=\frac{FW-DW}{FW}\times 100\%,$$ (2)

where FW and DW indicate the fresh and dry mass of each leaf (g), respectively, and *A* is the leaf area (cm^2^).

### Development of the new SAIs

**Fig 1:**
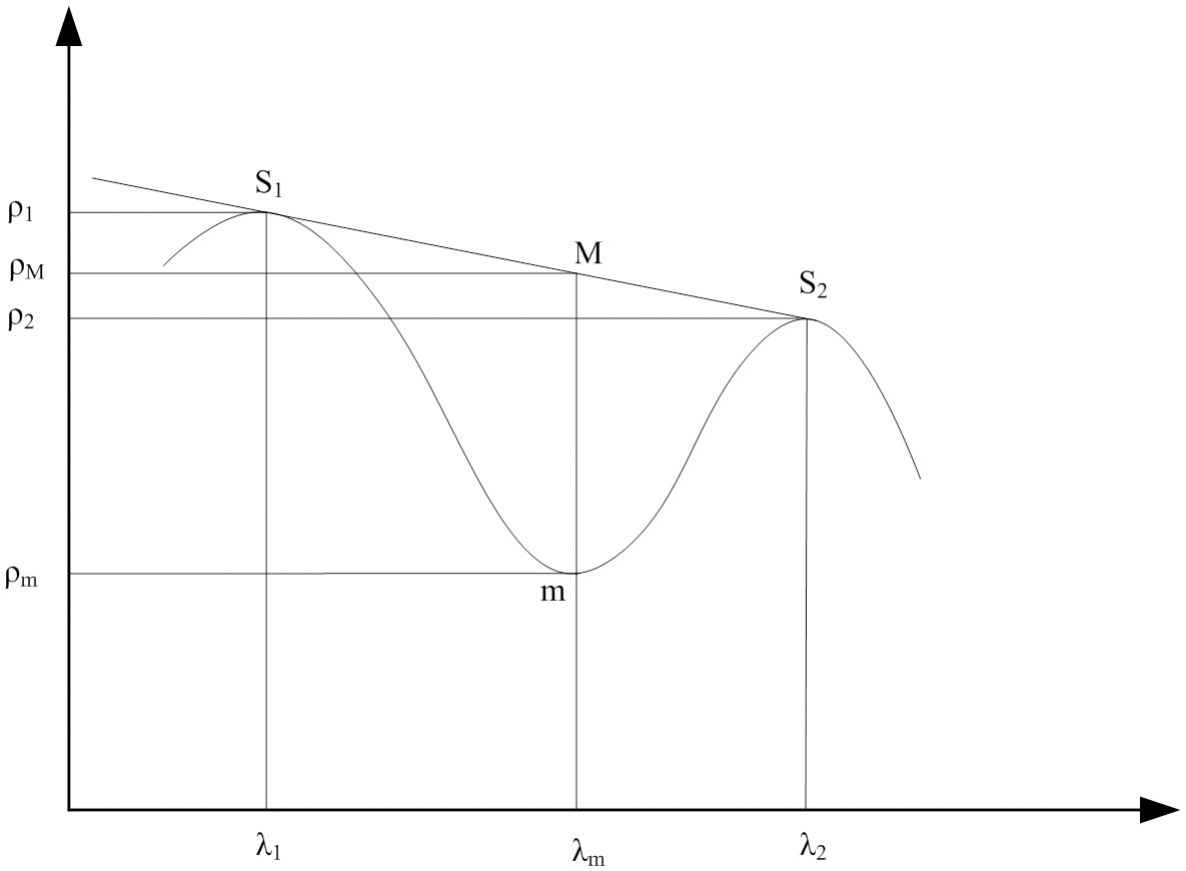
Spectral absorption feature. m is the Spectral Absorption Peak; S_1_ and S_2_ are Shoulders; *λ* is the Corresponding Wavelength; *ρ* is the Spectral Reflectance.

The spectral absorption feature was composed of the spectral absorption peak (wavelength position of the minimum reflectance of an absorption feature, point m in Fig 1) and two spectral absorption shoulder ends (S_1_ and S_2_ in Fig 1). The line between S_1_ and S_2_ was defined as the nonabsorption baseline, as shown in Fig 1. Wang et al. [49] defined the SAI as the ratio of the reflectance of the nonabsorption baseline at the wavelength position of the spectral band to that at the spectral absorption peak (SAI=ρMρm; *ρ*_*M*_ and *ρ*_m_ are shown in Fig 1).

According to the water absorption features and the SAI technique proposed by Wang et al. [49], we defined three new SAIs: SAI_970_, SAI_1200_, and SAI_1660_.

**Fig 2:**
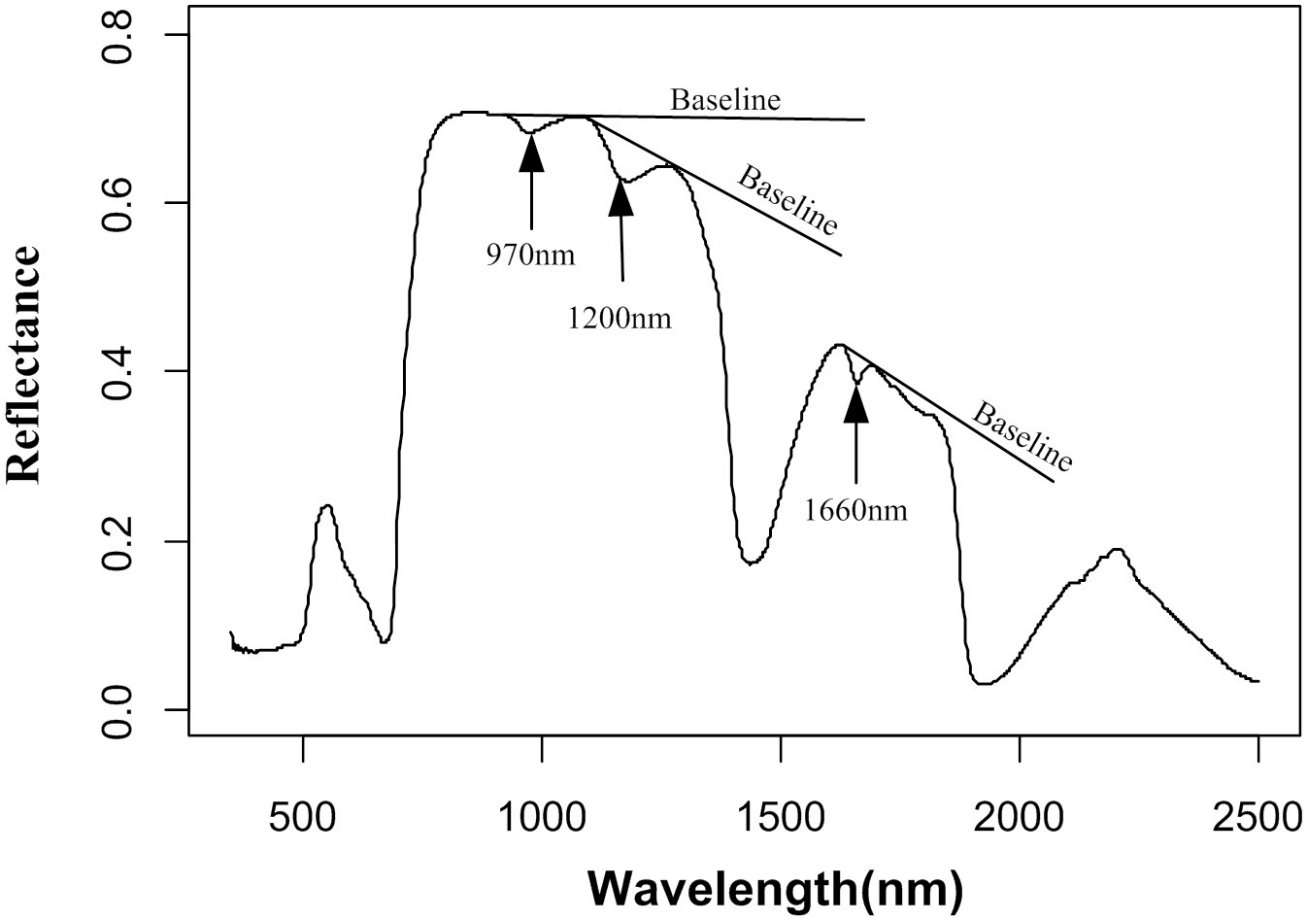
Typical reflectance spectrum of the sample (three absorption bands and nonabsorption baselines).

Fig 2 shows the three absorption bands at approximately 970 nm (915–1085 nm), 1200 nm (1085–1265 nm) and 1660 nm (1630–1690 nm) and the nonabsorption baseline formed by the shoulders of these bands, which were used to calculate the new SAIs in this study.

The three SAIs (SAI_970_, SAI_1200_, and SAI_1660_) were calculated using Eqs (3) and (4) [49], in which *d* is the absorption symmetry parameter (Eq (3)). The ten spectral indices based on the water absorption bands were calculated according to the formulas listed in Table 1.

$$d=(\lambda_{2}-\lambda_{m})/(\lambda_{2}-\lambda_{1}),$$ (3)

$$SAI=\frac{d\times \rho_{1}+(1-d)\times \rho_{2}}{\rho_{m}}.$$ (4)

**Table 1:** Spectral reflectance indices to estimate the water content and related source references.

Spectral index	Spectral index	Calculation formula	Reference
WI	Water Index	$\frac{\rho_{900}}{\rho_{970}}$	PeÑUelas et al. [23]
SRWI	Simple Ratio Water Index	$\frac{\rho_{858}}{\rho_{1240}}$	Zarco-Tejada et al. [42]
NDWI_1240_	Normalized Difference Water Index (NDWI)	$\frac{\left(\rho_{860}-\rho_{1240}\right)}{\left(\rho_{860}+\rho_{1240}\right)}$	Stimson et al. [35]
RDI	Relative Depth Index	$\frac{\left(\rho_{1116}\text{-min}(\rho_{1120-1150})\right)}{\rho_{1116}}$	Rollin and Milton [32]
RATIO_975_	Three-Band Ratio Index	$\frac{2{\bar{\rho }}_{960-990}}{\left({\bar{\rho }}_{920-940}+{\bar{\rho }}_{1090-1100}\right)}$	Pu et al. [24]
RATIO_1200_	Three-Band Ratio Index	$\frac{2{\bar{\rho }}_{1180-1220}}{\left({\bar{\rho }}_{1090-1110}+{\bar{\rho }}_{1265-1285}\right)}$	Pu et al. [24]
DWI	Depth Water Index	(*y*_1_ − *ρ*_970_)+(*y*_2_ − *ρ*_1200_)	Pasqualotto et al. [46]
MSI	Moisture Stress Index	$\frac{\rho_{1600}}{\rho_{820}}$	Hunt and Rock [43]
NDII	Normalized Difference Infrared Index	$\frac{\left(\rho_{860}-\rho_{1600}\right)}{\left(\rho_{860}+\rho_{1600}\right)}$	Hardisky et al. [44]
GVMI	Global Vegetation Moisture Index	$\frac{\left(\rho_{820}+0.1)-(\rho_{1600}+0.02\right)}{\left(\rho_{820}+0.1)+(\rho_{1600}+0.02\right)}$	Ceccato et al. [45]

*ρ* is the spectral reflectance; ${\bar{\rho }}_{\lambda_{1}-\lambda_{2}}$ is the average spectral reflectance in the *λ*_1_-*λ*_2_ region; min(*ρ*_1120–1150_) is the minimum spectral reflectance in the band range of 1120 nm to 1250 nm; and *y*_*i*_(*i* = 1,2) is calculated for *x*_*i*_(*x*_1_ = 970, *x*_2_ = 1200) as

$$y_{i}=\frac{\left(\rho_{1080}-\rho_{850}\right)}{230}*x_{i}+\frac{\left(\rho_{1080}*850-\rho_{850}*1080\right)}{-230}$$ (5)

Due to the differences in the vegetation species and internal structures, the absorption peaks or shoulders of all the leaves varied. Therefore, the key problem associated with the SAI calculations was to identify the absorption peak band (*λ*_*m*_ in Fig 1) and shoulders (*λ*_1_ and *λ*_2_ in Fig 1). In this study, the positions of the absorption peak and shoulders were obtained by calculating the minimum and maximum spectral reflectance values of each leaf in different bands by using R 4.0.1.

The performance of each index was tested using various fitting functions: linear, polynomial, and exponential functions.

### Validation strategy

To obtain robust results, the k-fold cross-validation method was used in this study. The basic principle of this technique can be described as follows [52]. First, the original dataset is divided into k subsets of approximately the same size. Second, the first dataset is used as the validation dataset, and the remaining k-1 datasets are combined to estimate the model parameters. Based on the model parameters, the dependent variables of the validation dataset are predicted, and the squared sum of the prediction errors is calculated. Third, the cross-validation process is repeated k times with each of the k subdatasets used as a validation dataset. In this study, we adopted a 10-fold (k = 10) cross-validation procedure.

The reliability of the indices for estimating the leaf EWT and FMC was evaluated considering the cross-validated coefficient of determination (*R*_*cv*_^*2*^) and relative cross-validated root mean square error (*rRMSE*_*cv*_). All the analyses were implemented in R 4.0.1. The *rRMSE*_*cv*_ was calculated using Eq (6).

$$rRMSE_{cv}=\frac{RMSE_{cv}}{\bar{EWT}or\bar{FMC}}$$ (6)

where $\bar{EWT}$ and $\bar{FMC}$ are the average values of the measured leaf EWT and FMC, respectively.

## Results

### Statistics of the measured plant variables

**Fig 3:**
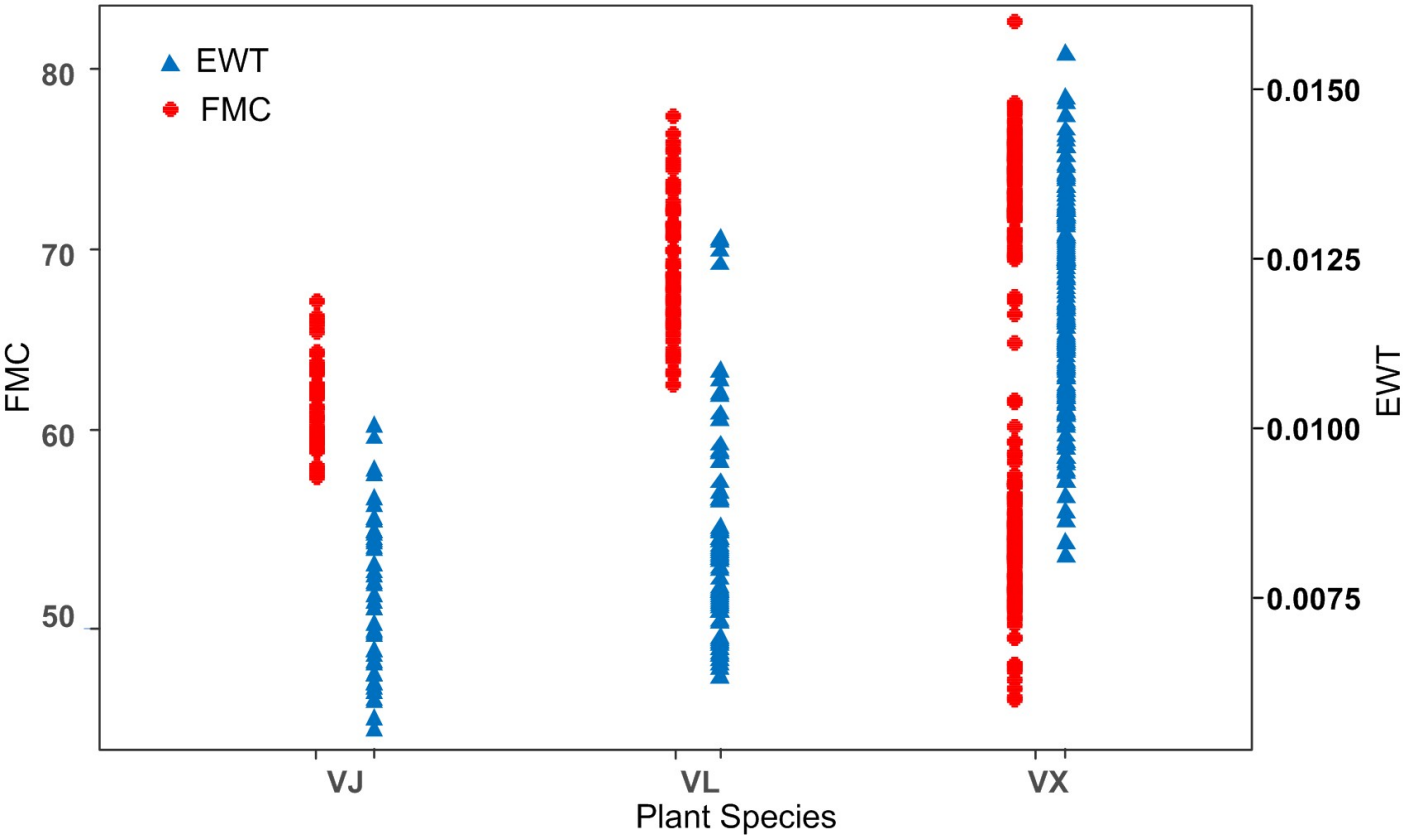
Leaf EWT (g cm^−2^) and FMC (%) of the three plant species.

In this study, 292 samples acquired from three plant species were used. Within the sample sites, the water content of leaves exhibited considerably variability: the EWT and FMC ranged from 0.006 g cm^**−2**^ to 0.016 g cm^**−2**^ and from 45.16% to 82.72% (Table 2), respectively. In addition, the leaf EWT and FMC among the three tree species were significantly different (Fig 3), with the highest EWT and FMC value of 0.016 and 82.72%, respectively, corresponding to VX and the lowest EWT and FMC values corresponding to VJ (0.006) and VX (45.16%).

**Table 2:** Statistics of the leaf EWT and FMC for the samples.

Species	Variable	Min	Max	Mean	Std. Deviation	N
Pooled data	EWT (g cm^−2^)	0.006	0.016	0.010	0.002	292
	FMC (%)	45.16	82.72	64.03	0.091	
VJ	EWT	0.006	0.010	0.008	0.001	46
	FMC	57.40	67.20	61.60	0.025	
VL	EWT	0.006	0.013	0.008	0.002	66
	FMC	62.60	77.50	**69.60**	0.036	
VX	EWT	0.008	0.016	**0.012**	0.001	180
	FMC	45.16	82.72	62.60	0.107	

### Retrieval of the EWT from the new SAIs

**Fig 4:**
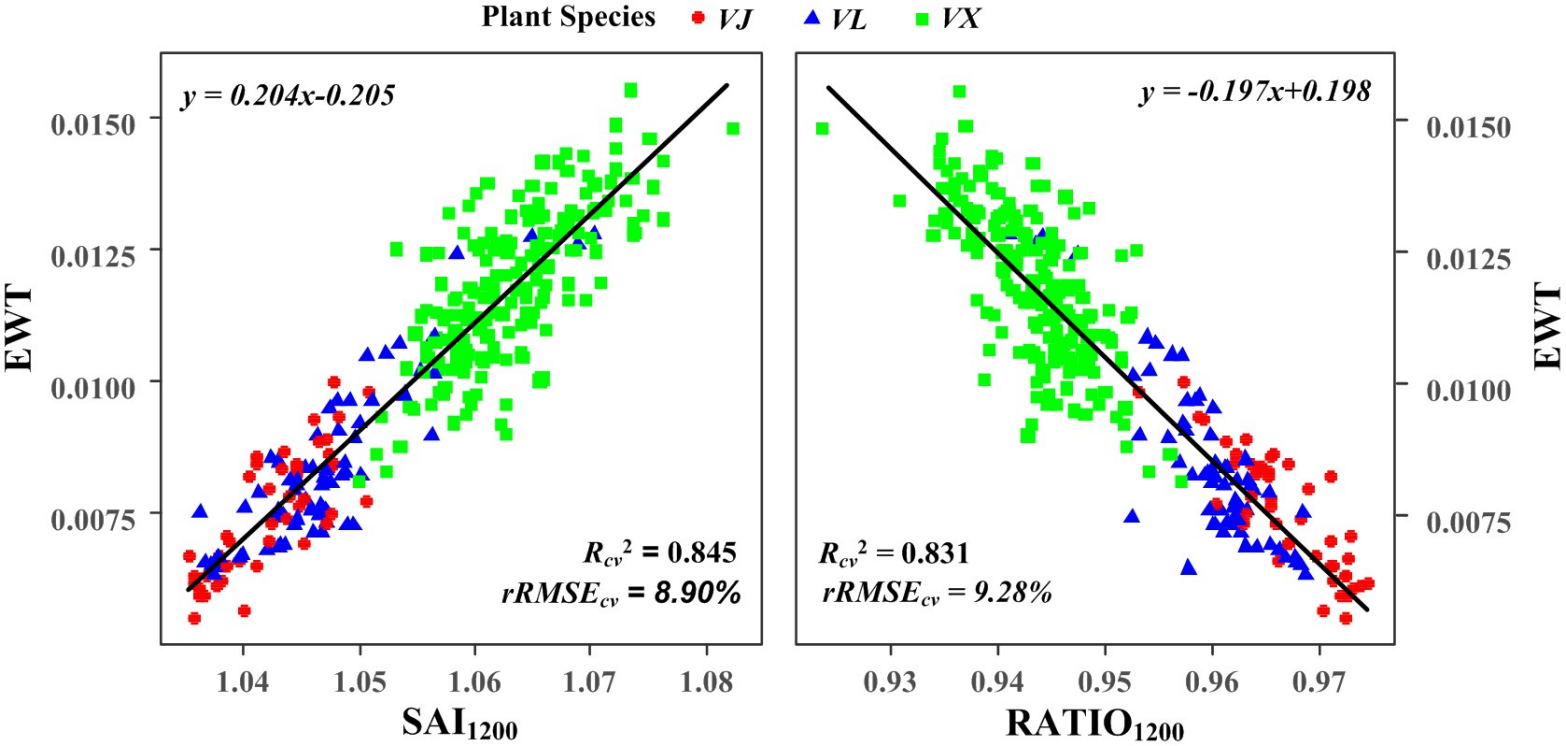
Relationships between the measured EWT and SAI_1200_ (left)/RATIO_1200_ (right) for the pooled data.

The leaf EWT was estimated using linear, polynomial and exponential functions. The results (Table 3) showed that (1) except for the RDI and RATIO_975_, all the indices exhibited a significant correlation with the EWT at the 0.01 level, even though the DWI and SAI_1660_ exhibited a less significant correlation with the EWT. (2) SAI_1200_, RATIO_1200_, the GVMI, the MSI and the NDII outperformed the other indices in estimating the EWT, with *R*_*cv*_^*2*^ values greater than 0.740 and *rRMSE*_*cv*_ values less than 11.41%. The optimal result was obtained using SAI_1200_, as indicated by the highest *R*_*cv*_^*2*^ of 0.845 and lowest *rRMSE*_*cv*_ of 8.90% in the linear fitting results (Fig 4), followed by that obtained using RATIO_1200_ with an *R*_*cv*_^*2*^ of 0.831 and an *rRMSE*_*cv*_ of 9.28% (Fig 4).

**Table 3:** Performance of the regression models in estimating the leaf equivalent thickness (EWT, g cm^−2^).

Index	EWT			
	P	Regression Equation	*Rcv*^*2*^	*rRMSEcv*
SAI_1200_	<0.0001	y = 0.204*x-0.205	0.845	8.90%
RATIO_1200_	<0.0001	y = -0.197*x+0.198	0.831	9.28%
GVMI	<0.0001	y = 0.042*x+0.001	0.76	11.04%
MSI	<0.0001	y = 0.033*x^2^-0.073*x+0.046	0.746	11.39%
NDII	<0.0001	y = 0.038*x+0.0038	0.744	11.41%
WI	<0.0001	y = 0.186*x-0.179	0.518	15.68%
NDWI	<0.0001	y = 0.762*x^2^+0.058*x+0.009	0.355	18.13%
SRWI	<0.0001	y = 0.165*x^2^-0.302*x+0.146	0.35	18.20%
SAI_970_	<0.0001	y = 0.100*x-0.093	0.25	19.68%
DWI	<0.0001	y = 0.032*x+0.007	0.113	21.27%
SAI_1660_	<0.001	y = -0.015*x+0.027	0.04	22.14%
RDI	n.s.	-	-	-
RATIO_975_	n.s.	-	-	-

*rRMSE*_*cv*_ is the Relative Cross-Validated Root Mean Square Error, and *R*_*cv*_^*2*^ is the Cross-Validated Coefficient of Determination.

n.s.: not significant at the 0.05 level (*n* = 292).

### Retrieval of the FMC from the new SAIs

**Fig 5:**
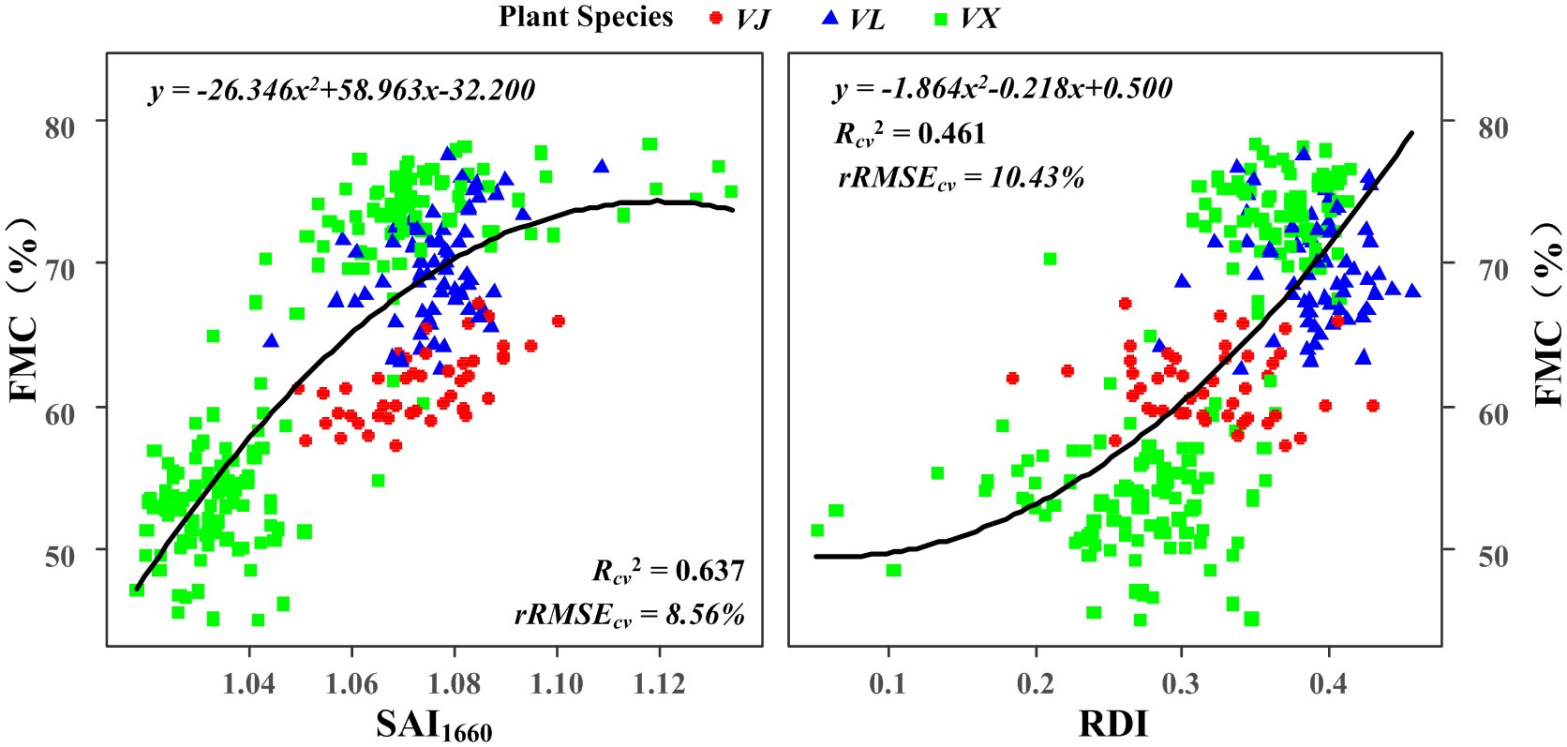
Relationships between the measured leaf FMC and SAI_1660_ (left)/RDI (right) for the pooled data.

The FMC was significantly correlated with all the indices except the GVMI and RATIO_1200_ at the 0.01 level for the pooled data (Table 4). SAI_1660_, SAI_970_, RATIO_975_, the RDI, and the DWI were more sensitive to the FMC than the EWT. SAI_1660_ achieved the optimal estimation of the FMC, as indicated by the highest *R*_*cv*_^*2*^ of 0.637 and lowest *rRMSE*_*cv*_ of 8.56% in the polynomial fitting results, followed by the RDI (*R*_*cv*_^*2*^ of 0.461 and *rRMSE*_*cv*_ of 10.43%) (Fig 5).

**Table 4:** Performance of the regression models for estimating the leaf FMC (%).

Index	FMC			
	P	Regression Equation	*Rcv*^*2*^	*rRMSEcv*
SAI1660	<0.0001	y = -26.346*x^2^+58.963*x-32.200	0.637	8.56%
RDI	<0.0001	y = -1.864*x^2^-0.218*x+0.500	0.461	10.43%
WI	<0.0001	y = 122.29*x^2^-242.87*x+125.15	0.32	11.73%
SAI970	<0.0001	y = 4.119*x-3.616	0.275	12.10%
DWI	<0.0001	y = -14.154*x^2^+4.520*x+0.345	0.235	12.43%
SAI1200	<0.0001	y = 297.64*x^2^-627*x+330.93	0.138	13.20%
RATIO975	<0.0001	y = -105.37*x^2^+200.88*x-95.061	0.135	13.22%
SRWI	<0.0001	y = 0.643*x-0.016	0.074	13.67%
NDWI	<0.0001	y = 1.289*x+0.627	0.071	13.69%
NDII	<0.0001	y = 6.791*x^2^-1.972*x+0.760	0.049	13.86%
MSI	<0.001	y = 2.804*x^2^-4.240*x+2.219	0.04	13.94%
RATIO1200	n.s.	-	-	-
GVMI	n.s.	-	-	-

*rRMSE*_*cv*_ is the Relative Cross-Validated Root Mean Square Error, and *R*_*cv*_^*2*^ is the Cross-Validated Coefficient of Determination.

n.s.: not significant at the 0.05 level (*n* = 292).

## Discussion

### Performance of the new SAIs in retrieving the water content

In this study, three new spectral absorption indices (SAI_970_, SAI_1200_, and SAI_1660_) were used to retrieve the leaf EWT and FMC from the reflectance spectra over three plant types dataset. To our knowledge, this study represents the first attempt to develop such SAIs for retrieving the leaf water content. The SAI is the ratio of the reflection intensity of the nonabsorption baseline at the wavelength position of the spectral band to that at the bottom of the spectral band. This ratio can also be defined as the "relative absorption depth".

As shown in Table 3, the EWT was positively correlated with SAI_1200_ and SAI_970_, but negatively correlated with SAI_1660_ for the pooled data. This phenomenon occurred because SAI_1200_ and SAI_970_ represent the relative absorption depth of the vegetation water near 970 nm and 1200 nm, respectively, and both indices are expected to increase with an increase in the leaf water content. In contrast, the absorption characteristic near 1660 nm is related to the leaf dry matter constituents (e.g. lignin and cellulose) that become prominent as the water content decreases [29, 53]. This aspect also explains why SAI_1660_ was more closely related to the mass based parameter FMC than the area based parameter EWT. According to this principle, FMC was more likely to be species dependent than EWT, as discussed in the following section.

Among the indices, the new spectral absorption index SAI_1200_ was the most suitable index for estimating the EWT at the leaf level, as indicated by the *R*_*cv*_^*2*^ of 0.845 and *rRMSE*_*cv*_ of 8.90%. However, SAI_970_ achieved only a moderate accuracy in predicting the EWT at the leaf level. The results were consistent with those reported by Kovar et al. [5] who indicated that compared to that at 1200 nm, the absorption characteristic at 970 nm showed relatively weaker sensitivity to the leaf EWT in their study on soybean plants. This phenomenon likely occurred because the absorption characteristic of water at 970 nm is weaker than that at 1200 nm; moreover, the reflectance at 970 nm is more significantly affected by the vegetation structure and other factors (leaf structure and dry matter content) than that at 1200 nm [47]. This aspect is likely why SAI_970_ exhibits a slightly stronger correlation with FMC than SAI_1200_, because FMC is a mass based parameter and has a stronger correlation with dry matter than EWT.

In terms of the traditionally simple or normalized ratio index configured with only a few specific spectral bands, significant relationships were observed between the EWT and GVMI, MSI, NDII (*R*_*cv*_^*2*^ > 0.70), while weaker correlations were achieved with WI, NDWI, SRWI (*R*_*cv*_^*2*^ < 0.55). The results are consistent with those of other studies, which indicated that the spectral indices involving the combination of the SWIR and NIR wavelengths were more effective to estimate the leaf EWT than those that only combined the NIR wavelengths [33, 47, 54]. However, compared with SAI_1200_ and RATIO_1200_, these indices were suboptimal, and SAI_1200_ and RATIO_1200_ combined only the NIR wavelengths. The results demonstrate that selecting an appropriate vegetation index in the NIR bands can effectively indicate the change in the leaf water content, especially among the indices derived from the absorption feature bands near 1200 nm. This phenomenon occurs because more notable water absorption characteristic exist near 1200 nm than at 970 nm [6].

The data in Table 4 show that SAI_1660_ is the most strongly correlated with the leaf FMC, as indicated by the *R*_*cv*_^*2*^ of 0.637 and *rRMSE*_*cv*_ of 8.56%. The other indices except for the GVMI and RATIO_1200_ are only slightly related to the leaf FMC (*R*_*cv*_^*2*^ *< 0*.*50*). Our results confirm that the traditionally spectral indices are more suitable for estimating the EWT than the FMC at the leaf level [33, 54].

Furthermore, the results demonstrate that SAI_1200_ and SAI_1660_ can represent the variation characteristics of the spectral absorption features and help estimate the leaf water content. The high performance of the SAI_1200_ and SAI_1660_ can be attributed to the fact that the SAIs were constructed considering the symmetry in the absorption characteristics and spectral reflectance heterogeneity of different leaves; moreover, the SAI could eliminate the spectral contribution of the nonabsorbent materials through the nonabsorption baseline equation and ratio processing and measure the relative spectral absorption depth of water or dry matter components.

### Comparison of the SAIs and RATIO indices

#### Similarity in the SAIs and RATIO indices

The methods to establish the SAIs (SAI_970_ and SAI_1200_) and RATIO indices (RATIO_975_ and RATIO_1200_) were similar, and the values were obtained by calculating the ratio of the absorption bands near 970 nm and 1200 nm and the corresponding absorption shoulder bands. Both SAI_970_ and RATIO_975_ were more sensitive to the FMC than the EWT, whereas SAI_1200_ and RATIO_1200_ were more sensitive to the EWT.

**Fig 6:**
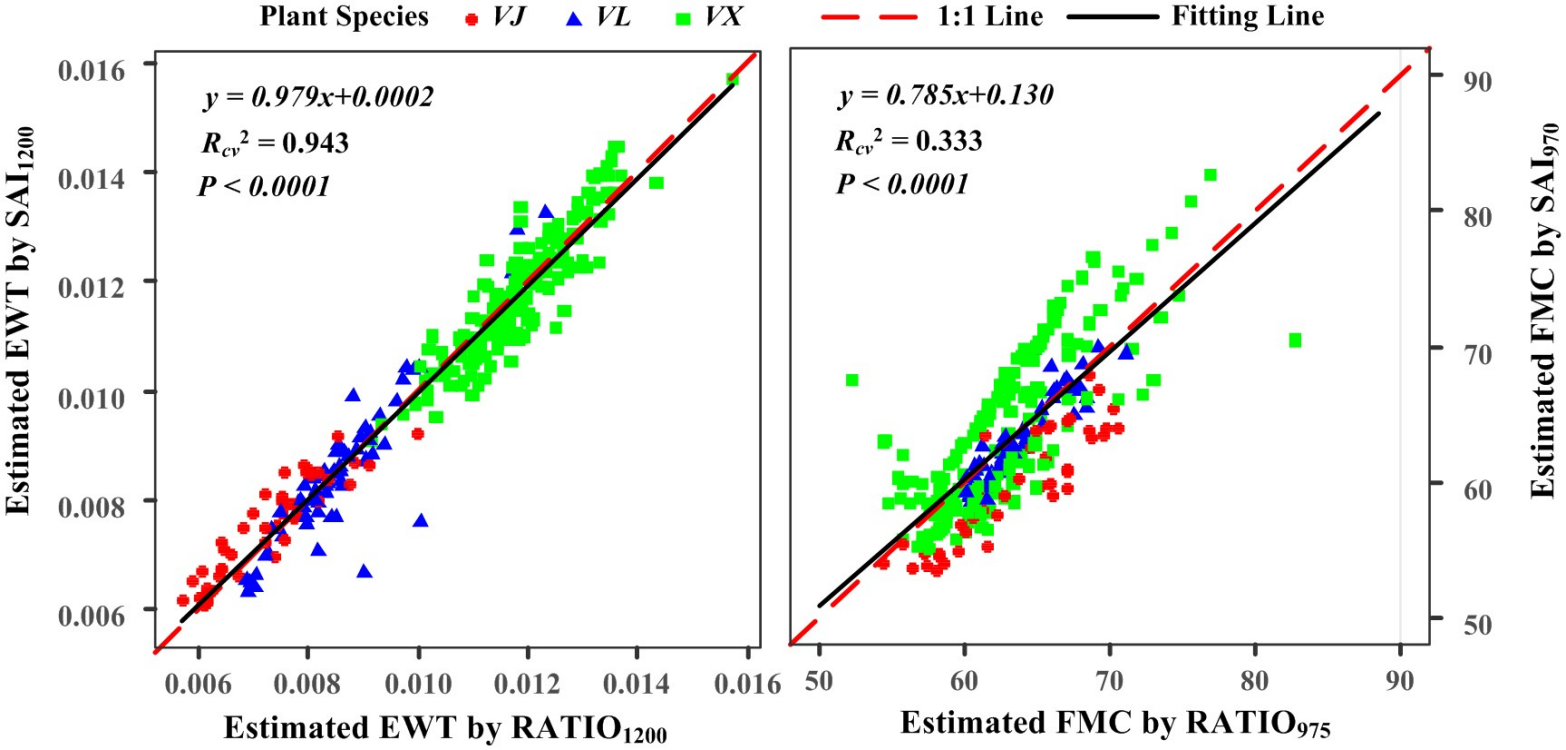
Plots of the EWT and FMC estimated using RATIO and SAI for the pooled data.

To demonstrate the similarity, the FMC estimated using SAI_970_ and EWT estimated using SAI_1200_ were plotted against the FMC values estimated using RATIO_975_ and EWT values estimated using RATIO_1200_ (Fig 6). The similarity between SAI_1200_ and RATIO_1200_ was more notable than that between SAI_970_ and RATIO_975_, as indicated by the *R*_*cv*_^*2*^ values of 0.943 and 0.333, respectively. This difference might be interpreted as follows. The reflectivity of the absorption band at 970 nm was more significantly affected by other factors (leaf structure and dry matter content) than that of the absorption band at 1200 nm, thereby increasing the difference in the reflectivity of the absorption band at 970 nm among different leaves [47]. Therefore, the similarity between RATIO_975_ calculated with the same three specific bands for all leaves and SAI_970_ calculated with the absorption peak and shoulders of individual leaves was not significant. In other words, the absorption characteristics at 970 nm were more susceptible to other factors, including water, than those at the absorption band at 1200 nm.

### Superiority of the SAIs over the RATIO indices

In this study, the SAIs (SAI_970_ and SAI_1200_) could more accurately estimate the FMC and EWT than the RATIO indices (RATIO_975_ and RATIO_1200_) (Table 2) since the SAIs were constructed considering the symmetry in the absorption characteristics and spectral reflectance heterogeneity of different leaves, which were not considered when constructing the RATIO indices. Moreover, the SAIs were calculated considering the absorption peak and absorption shoulder band of each leaf, whereas the RATIO indices were calculated considering the average spectral reflectance in a specific band range. Pu et al. [29] reported that the absorption position shifted to shorter wavelengths at 975 nm and 1200 nm and to a longer wavelength at 1750 nm as the leaf water content increased. However, using the average value in a specific band range as the value of the absorption feature peaks or troughs may obscure or weaken the change in the leaf spectral absorption feature induced by the water content.

### Inversion of the SAIs and RATIO indices for the leaf FMC

Pu et al. [29] found that RATIO_975_ and RATIO_1200_ outperformed the indices derived from the band at 1750 nm in evaluating the FMC. In our study, we obtained the opposite results. SAI_1660_ outperformed the other indices in evaluating the FMC, as indicated by the *R*_*cv*_^*2*^ of 0.637 and an *rRMSE*_*cv*_ of 8.56% in this study. However, RATIO_975_ was weakly correlated with the FMC (*R*_*cv*_^*2*^ of 0.135 and *rRMSE*_*cv*_ of 13.22%), and even at the 0.05 level, the correlation between RATIO_1200_ and the FMC was not significant. The different results may be caused by the species differences because the water absorption band centered at 1750 nm (1650–1850 nm) is an indirect absorption band and is ascribed to chemicals such as cellulose, sugar and starch [29]. In addition, Pu et al. [29] analyzed the leaves of specific plant species (coastal live oak), whereas we used a multiplant dataset pooled with three plant species, specifically, camphor (VX), capricorn (VJ), and red-leaf plum (VL) trees.

### Dependency of the new SAIs on the plant species

**Fig 7:**
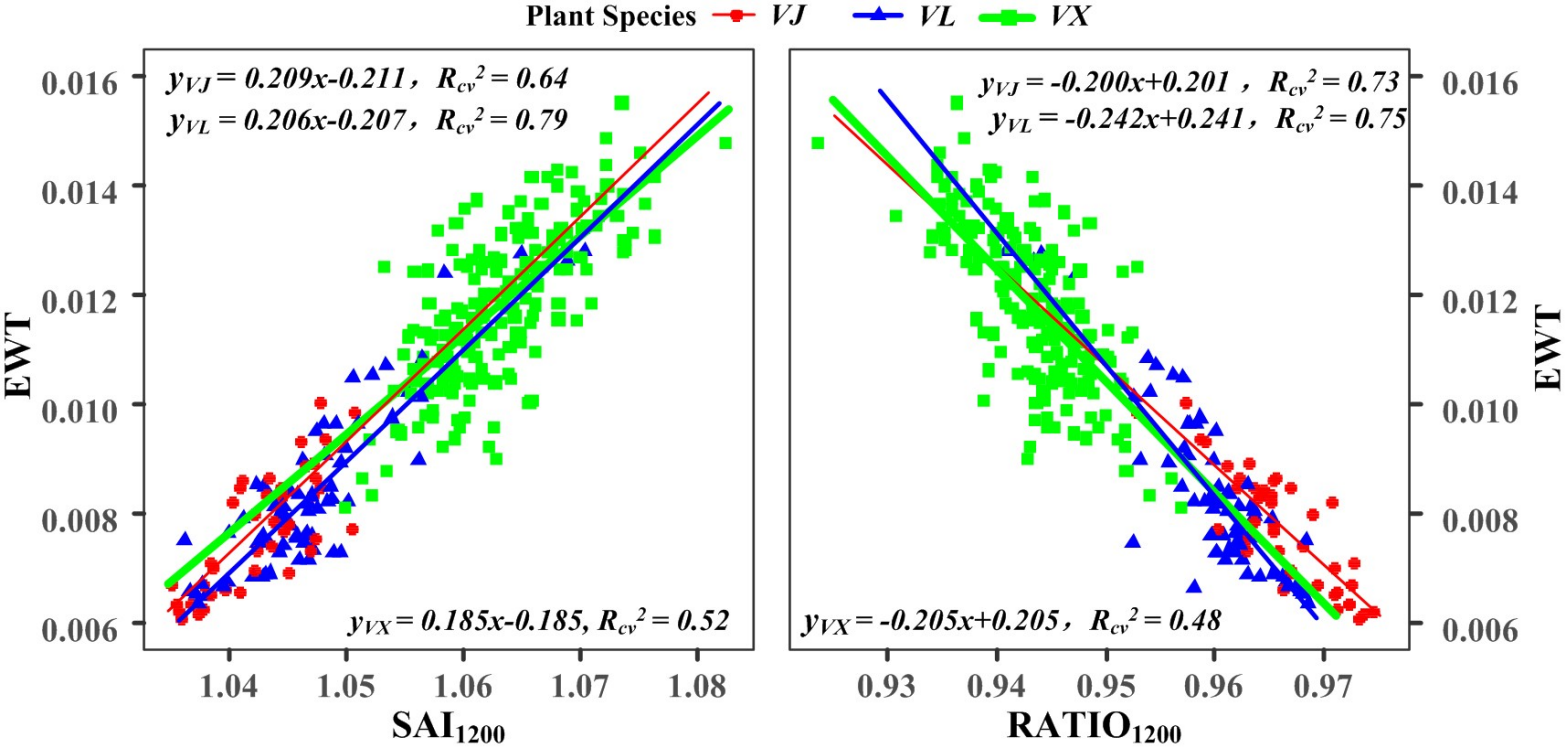
Relationship between the measured EWT and SAI_1200_ (left) and RATIO_1200_ (right) at the species level.

Considering the influence of the plant species on the estimation of the water content parameters, we estimated the EWT of the three plant species considering SAI_1200_ and RATIO_1200_ (Fig 7). The estimation performances of SAI_1200_ and RATIO_1200_ for the three plant species were similar (Fig 7). The slopes of the linear regression lines for three plant species obtained with SAI_1200_ ranged from 0.185 (VX) to 0.209 (VJ), and the intercept ranged from -0.211 (VJ) to -0.185 (VX). The ranges of the slope and intercept were 0.024 and 0.026, respectively.

**Fig 8:**
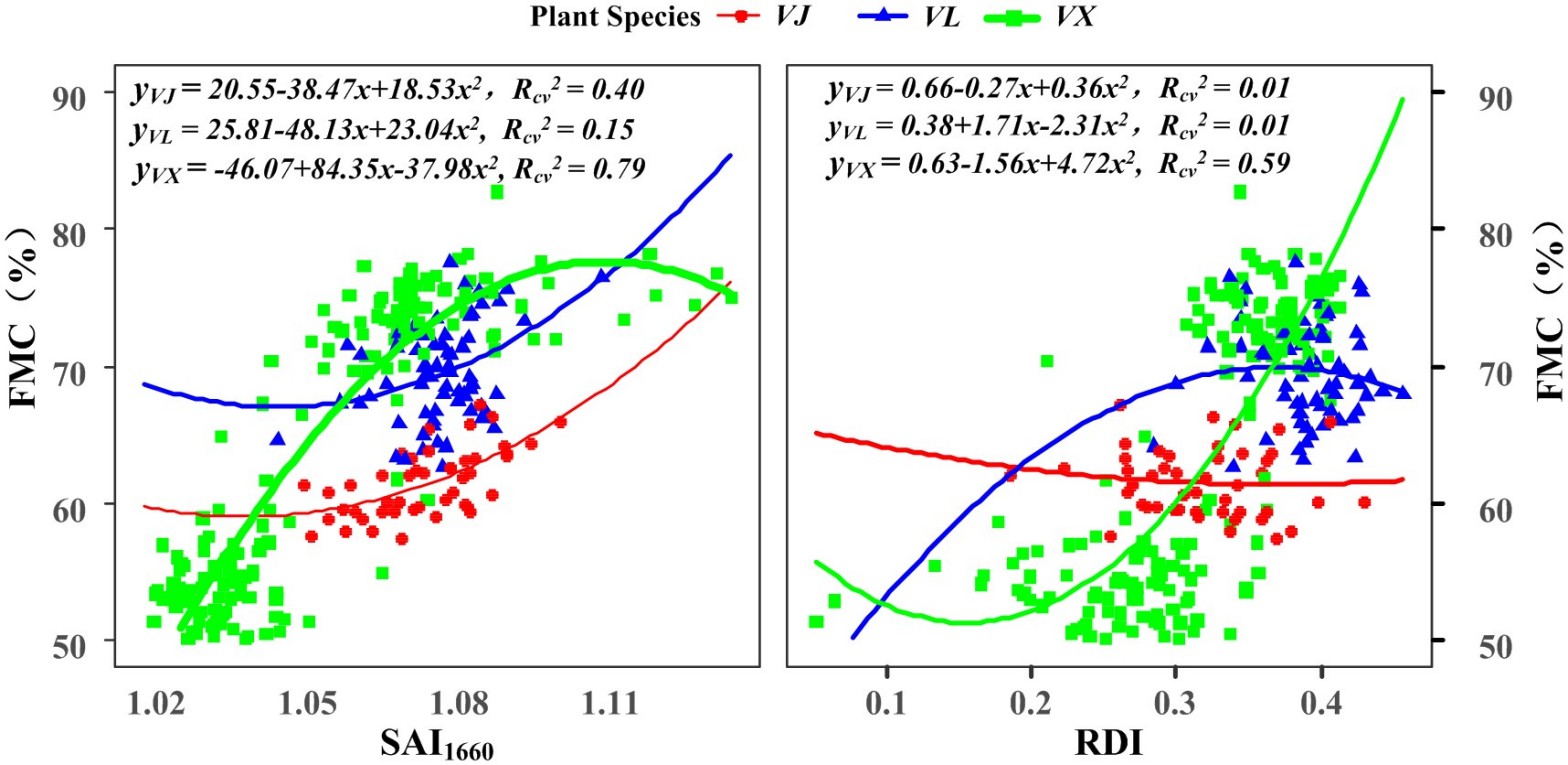
Relationship between the measured FMC and SAI_1660_ (left) and RDI (right) at the species level.

The FMC of the three plant species was estimated using SAI_1660_ and the RDI (Fig 8), and it was noted that the estimation performances of SAI_1660_ and the RDI for the three plant species were considerably different (e.g., the quadratic coefficient of the quadratic equation for the three plant species obtained using SAI_1660_ ranged from -37.98 (VX) to 23.04 (VL), and the coefficient of the primary term ranged from 84.35 (VX) to -48.13 (VL)). Expectedly, VX obtained the highest accuracy of leaf FMC estimation when using SAI_1660_, followed by VJ. This phenomenon occurred because the spectral absorption characteristics of vegetation near the wavelength of 1660 nm become more notable with the decrease in the leaf FMC [29, 53]. Among the three species, the average FMC of VL was the highest (69.60%), and the corresponding values for VX (62.6%) and VJ (61.6%) were lower. Although the average FMC of VX was similar to that of VJ (or even slightly higher), VX could more accurately estimate the leaf FMC using SAI_1660_, likely because of the wider changes in the FMC of VX (45.16% to 82.72%) than that of VJ (57.40% to 67.20%) and the larger sample size of the former parameter.

The correlation between the EWT and spectral indices from the pooled data was more significant than that between the EWT and those obtained from the data of individual species, as indicated by the *R*_*cv*_^*2*^ of 0.845 and 0.831 for SAI_1200_ and RATIO_1200_, respectively (Figs 4 and 7). However, the correlation between the FMC and spectral indices from the pooled data was not more significant than that between the FMC and those obtained from the data of the individual species (Figs 5 and 8). These phenomena indicated that the observed relationships between the FMC and reported spectral indices were more likely to be species specific than those between the EWT and spectral indices (SAI_1200_ and RATIO_1200_). In other words, the leaf EWTs estimated using the spectral indices (SAI_1200_ and RATIO_1200_) were less influenced by variations in the internal leaf structures than the leaf FMCs estimated using the spectral indices (SAI_1660_ and RDI).

### Importance of the FMC in estimating the leaf growth

The FMC is a mass based parameter. Pu et al. [24] noted that the data points of fresh green leaves and brown-gray leaves form two clusters in the scatter plots of several spectral features with the FMC. A similar phenomenon was observed in this study. A notable gap existed between the new leaves (green square dots in the top region in Fig 8) and mature leaves (green square dots in the bottom region in Fig 8) of the same plant species (VX), although this gap was not reflected in the EWT (Fig 7). This finding further confirmed that the mass based FMC parameter is important for estimating the leaf growth [16]. We speculate that the FMC might be more suitable for distinguishing the leaf water status at different growth stages than the EWT. To verify this aspect, additional work is necessary such as that involving the collection of leaf samples from different seasons and more plant species.

### Uncertainties

The SAI was compared with commonly used water vegetation indices; however, the corresponding approach was not compared with other methods (e.g., the use of derivative spectra and indicators after continuum removal and similarity matrix and artificial neural network methods). In addition, this study considered only three common vegetation types in the same season, and thus, the applicability of these indices to other vegetation types in different growing seasons must be examined in the future. Nevertheless, our research results provide a basis for subsequent research and confirm the application potential of SAIs in vegetation water retrieval. Moreover, this study demonstrates a novel concept for the hyperspectral inversion of vegetation water.

## Conclusions

Considering the symmetry of spectral absorption at 970 nm, 1200 nm and 1660 nm and spectral heterogeneity of different leaves, we proposed three new SAIs (SAI_970_, SAI_1200_, and SAI_1660_) to retrieve the leaf EWT and FMC for various plant types. The following key conclusions were derived: (1) SAI_1200_ was more suitable for estimating the EWT, whereas SAI_970_ and SAI_1660_ were more suitable for estimating the FMC. (2) SAI_970_ and SAI_1200_ outperformed RATIO_975_ and RATIO_1200_, respectively, in estimating the FMC and EWT. (3) The new SAIs (SAI_1200_ and SAI_1660_) can effectively estimate the leaf water content.

## Supporting information

S1 File (PDF)

S2 File (PDF)

S1 Data (CSV)
